# Methylphenidate remediates aberrant brain network dynamics in children with attention-deficit/hyperactivity disorder: A randomized controlled trial

**DOI:** 10.1016/j.neuroimage.2022.119332

**Published:** 2022-05-28

**Authors:** Yoshifumi Mizuno, Weidong Cai, Kaustubh Supekar, Kai Makita, Shinichiro Takiguchi, Akemi Tomoda, Vinod Menon

**Affiliations:** a Department of Psychiatry & Behavioral Sciences, Stanford University School of Medicine, Stanford, CA 94304, USA; b Research Center for Child Mental Development, University of Fukui, Fukui, 910-1193, Japan; c Division of Developmental Higher Brain Functions, United Graduate School of Child Development, University of Fukui, Fukui, 910-1193, Japan; d Department of Child and Adolescent Psychological Medicine, University of Fukui Hospital, Fukui, 910-1193, Japan; e Department of Neurology and Neurological Sciences, Stanford University, Stanford, CA 94304, USA; f Wu Tsai Neurosciences Institute, Stanford University, Stanford, CA 94304, USA; g Maternal & Child Health Research Institute, Stanford University, Stanford, CA 94304, USA

**Keywords:** Methylphenidate, Attention-deficit/hyperactivity disorder, Dynamic time-varying cross-network, interaction, Cognitive control network, Salience network

## Abstract

Methylphenidate is a widely used first-line treatment for attention deficit/hyperactivity disorder (ADHD), but the underlying circuit mechanisms are poorly understood. Here we investigate whether a single dose of osmotic release oral system methylphenidate can remediate attention deficits and aberrancies in functional circuit dynamics in cognitive control networks, which have been implicated in ADHD. In a randomized placebo-controlled double-blind crossover design, 27 children with ADHD were scanned twice with resting-state functional MRI and sustained attention was examined using a continuous performance task under methylphenidate and placebo conditions; 49 matched typically-developing (TD) children were scanned once for comparison. Dynamic time-varying cross-network interactions between the salience (SN), frontoparietal (FPN), and default mode (DMN) networks were examined in children with ADHD under both administration conditions and compared with TD children. Methylphenidate improved sustained attention on a continuous performance task in children with ADHD, when compared to the placebo condition. Children with ADHD under placebo showed aberrancies in dynamic time-varying cross-network interactions between the SN, FPN and DMN, which were remediated by methylphenidate. Multivariate classification analysis confirmed that methylphenidate remediates aberrant dynamic brain network interactions. Furthermore, dynamic time-varying network interactions under placebo conditions predicted individual differences in methylphenidate-induced improvements in sustained attention in children with ADHD. These findings suggest that a single dose of methylphenidate can remediate deficits in sustained attention and aberrant brain circuit dynamics in cognitive control circuits in children with ADHD. Findings identify a novel brain circuit mechanism underlying a first-line pharmacological treatment for ADHD, and may inform clinically useful biomarkers for evaluating treatment outcomes.

## 1. Introduction

Attention-deficit/hyperactivity disorder (ADHD) is one of the most commonly diagnosed neurodevelopmental disorders in childhood (American Psychiatric Association 2013, Wolraich et al., 2019). Clinical symptoms of ADHD include inattention, hyperactivity, and impulsivity, which are thought to arise from dysfunctional attention and cognitive control circuits (Cai et al., 2018, Sripada et al., 2014, Posner et al., 2020). The adverse consequences of ADHD often persist through adolescence into adulthood, leading to academic, social, and employment difficulties (Wolraich et al., 2019). Early treatment of ADHD is therefore critical for improving cognitive and behavioral outcomes in affected children.

Methylphenidate is a stimulant that is widely used as a first-line medication for the treatment of ADHD (McLennan, 2016), and has been shown to improve cognitive performance in children with ADHD (Mueller et al., 2017), and ameliorate inattention, hyperactivity, and impulsivity symptoms (Schachter et al., 2001). However, about 30% of children with ADHD do not respond to methylphenidate, and there are no reliable predictors of individual patient responses (Van der Oord et al., 2008, Wilens, 2008, Faraone et al., 2015). Brain imaging studies have showed that methylphenidate alters frontal and parietal cortex activation associated with performance of sustained attention and inhibitory control tasks (Kowalczyk et al., 2019, Czerniak et al., 2013). Impairments in large-scale brain circuits are now recognized as prominent neurobiological signatures of ADHD (Posner et al., 2014, Castellanos and Aoki, 2016). However, the brain circuit mechanisms by which methylphenidate remediates ADHD symptoms and cognitive deficits are poorly understood. Understanding the effects of methylphenidate on functional brain circuits associated with cognitive control is critical for elucidating the pathophysiology of ADHD, and has the potential to inform sources of individual differences in treatment response, as well as the development of robust predictors of clinical course which would aid treatment decisions (Posner et al., 2020).

Brain systems involved in cognitive control are important targets for the investigation of functional circuit mechanisms by which methylphenidate remediates attentional deficits. These include, most prominently, the salience network (SN), frontoparietal network (FPN) and default mode network (DMN), which play a crucial role in virtually all tasks that require moment-by-moment changes in adaptive cognitive control (Menon and Uddin, 2010, Menon, 2015, Menon, 2011). The SN, which is anchored in the anterior insula and anterior cingulate cortex, is important for identifying biologically and cognitively salient events necessary for guiding attention and goal-directed behaviors (Menon and Uddin, 2010). The FPN, which is anchored in the dorsolateral prefrontal cortex and the posterior parietal cortex, is involved in the active maintenance and manipulation of information in working memory (Owen et al., 2005). Finally, the DMN, which is anchored in the posterior cingulate cortex and the medial prefrontal cortex, plays a critical role in self-referential mental processes (Gusnard et al., 2001). Critically, disturbances in these cognitive control networks are a prominent feature of childhood ADHD (Posner et al., 2014, Castellanos and Aoki, 2016, Cai et al., 2019). Altered time-averaged intrinsic connectivity within the DMN, between the SN and DMN, and between the SN and FPN have been identified in ADHD (Sripada et al., 2014, Sutcubasi et al., 2020). More broadly, the triple-network model of cognitive dysfunction in psychopathology (Menon and Uddin, 2010, Menon, 2015) posits a central role for the SN in initiating switching between the FPN and DMN, a process essential for attention and flexible cognitive control (Cai et al., 2016, Chen et al., 2015, Supekar and Menon, 2012). Based on evidence that attention and cognitive control relies on dynamic cross-network interactions (Braun et al., 2015, Chen et al., 2016, Taghia et al., 2018), we previously examined SN-mediated dynamic time-varying cross-network interactions with FPN and DMN in children with ADHD, and found these interactions were aberrant in children with ADHD in two independent cohorts (Cai et al., 2018). The central question of the present study was whether methylphenidate administration remediates aberrant functional brain circuit dynamics between the SN, FPN, and DMN in children with ADHD.

**Fig. 1.**
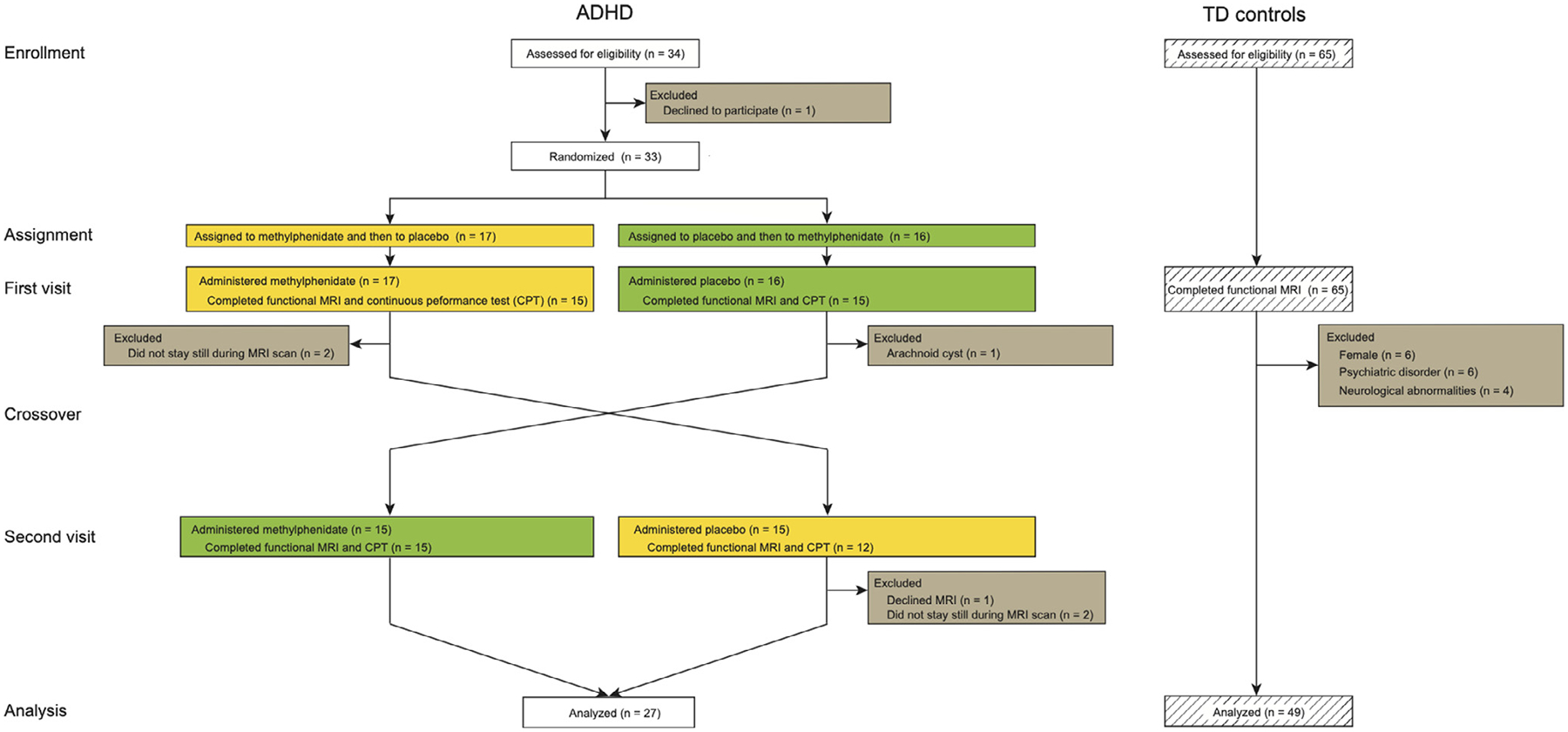
Study design. Randomized placebo-controlled double-blind crossover design to investigate the brain circuit mechanisms that underlie the therapeutic effects of a single dose of osmotic release oral system methylphenidate administration in children with attention-deficit/hyperactivity disorder (ADHD). CPT, continuous performance task; MRI, magnetic resonance imaging; TD, typically-developing.

Here we use a randomized placebo-controlled double-blind crossover design (Fig. 1) to investigate the effect of methylphenidate on dynamic functional brain circuit between the SN, FPN and DMN in children with ADHD. Dynamic brain circuit measures were examined under both methylphenidate and placebo conditions in 27 children with ADHD, and contrasted with baseline data from 49 typically-developing (TD) children. We first test the hypothesis that methylphenidate remediates sustained attention deficit in children with ADHD. We then test the hypothesis that methylphenidate remediates aberrant dynamic time-varying cross-network interactions between the SN, FPN and DMN. Finally, we explore whether dynamic time-varying cross-network interactions under placebo conditions can predict the effects of methylphenidate on sustained attention.

## 2. Materials and methods

### 2.1. Study design and participants

This study protocols were approved by the Ethics Committee of the University of Fukui (Assurance no. 20170005). All participants and the parent(s) provided written informed consent and assent for participation in this study. This study is registered with the University Hospital Medical Information Network (UMIN000027533).

Fig. 1 shows the overall study design (see Supplemental Methods for details). 34 children with ADHD were recruited at the University of Fukui Hospital, Japan, and 65 TD children were recruited from the community. ADHD diagnosis was based on the Diagnostic and Statistical Manual of Mental Disorders, Fifth Edition (DSM-5), and was confirmed in structured interviews with investigators using the Japanese Version of the Kiddie Schedule for Affective Disorders and Schizophrenia for School-Aged Children-Present and Lifetime Version (K-SADS-PL-J) (Kaufman et al., 1997). Inclusion criteria for both groups were no contraindications for magnetic resonance imaging (MRI), full scale intelligence quotient (FSIQ) > 70, no history of severe head trauma or neurological abnormalities (e.g. epilepsy, arachnoid cysts). To minimize the potential impact of sex differences we included only male participants, consistent with the male bias in the prevalence of ADHD (Willcutt, 2012, Xu et al., 2018). Participants with excessive head motion (over 3.0 mm, 3.0 degree, and mean framewise displacement (FD) 0.3 mm) during the scanning were excluded (Mizuno et al., 2017). All participants were medication-free prior to MRI for at least 5 times half-lives, including methylphenidate and atomoxetine, consistent with protocols from previous studies (Mizuno et al., 2017, Fair et al., 2010).

Children with ADHD were scanned twice, in a randomized placebo-controlled double-blind crossover design. During the first visit, they were administered a single dose of osmotic release oral system methylphenidate (OROS-MPH) (1.0mg/kg: 1.0 ± 0.1mg/kg) or placebo (lactose) under double-blind conditions, as previous studies (Akhondzadeh et al., 2004, Wilens et al., 2005). We used OROS-MPH rather than immediate release methylphenidate because immediate release methylphenidate is not approved for clinical use in Japan. Five to eight hours after administration, when methylphenidate concentration in the blood is maximal (American Psychiatric Association 2007), they underwent a resting-state functional MRI (fMRI) scan. They also performed a standardized continuous performance task (CPT) (Huang-Pollock et al., 2012, Shin et al., 2000), outside the MRI scanner to evaluate sustained attention.

During the second visit, within 1 to 6 weeks after the first visit, they underwent a resting-state fMRI scan and performed the CPT after they took the second medicine: children with ADHD who took a single dose of OROS-MPH at the first visit took the placebo at the second visit under double-blind conditions, and vice versa. OROS-MPH condition was defined as ADHD-MPH, and placebo condition was defined as ADHD-Placebo in this study. TD controls were scanned once without OROS-MPH or placebo, and the CPT was not administered to the TD controls. Children with ADHD took their regularly prescribed medications between the two visits, but stopped medication prior to each MRI session, as described above.

### 2.2. Sustained attention

A standardized CPT was administered to children with ADHD under both the methylphenidate and placebo conditions. The task consisted of a Go/NoGo paradigm in which children were presented with either a target or non-target stimulus on the screen for 100 msec, once every 2 seconds for 15 minutes across three 5-minute blocks. The target stimulus was a triangle, while the non-target stimulus was either a circle or a square. Children were required to press the button when a target stimulus was presented, and withhold response to non-targets. The percentage of target stimuli varied between 22%, 50% and 78% across blocks (Fujioka et al., 2016). The test has been normed with age-adjusted T-scores on four distinct performance measures: omission errors (failing to respond to targets), commission errors (false response to non-targets), mean response time (RT), and standard deviation of RT (Huang-Pollock et al., 2012, Shin et al., 2000). To further evaluate overall performance on the CPT, we then computed a composite performance score by averaging standardized scores of omission error, commission error, mean RT, and standard deviation of RT in the continuous performance task, with lower scores reflecting better performance.

### 2.3. fMRI data acquisition

Functional images were acquired with a T2*-weighted gradient-echo echo-planar imaging (EPI) sequence via a 3-T scanner (Discovery MR 750; General Electric Medical Systems, Milwaukee, WI) and a 32-channnel head coil. In total, 201 volumes were acquired for a total scanning time of 7 minutes 42 seconds. Each volume consisted of 40 slices, with a thickness of 3.5 mm and a 0.5-mm gap to cover the entire brain. The time interval between each successive acquisition of the same slice (repetition time, TR) was 2300 ms, with an echo time (TE) of 30 ms, and a flip angle (FA) of 81°. The field of view (FOV) was 192 × 192 mm, and the matrix size was 64 × 64, yielding volume dimensions of 3 × 3 mm. The participants were instructed to stay awake but close their eyes and think of nothing in particular. Participant movement was further minimized by the placement of memory-foam pillows around their head, as previously reported (Mizuno et al., 2017, Jung et al., 2015).

### 2.4. fMRI data pre-processing and ICA analysis

A standard preprocessing procedure was implemented using SPM12 (http://www.fil.ion.ucl.ac.uk/spm/), including slice-timing correction, realignment, normalization, spatial smoothing (6-mm smoothing kernel), regression of nuisance variables (24 motion parameters, white matter, and cerebrospinal fluid signals), and bandpass filtering (0.008 Hz < f < 0.1Hz) (Cai et al., 2018, Supekar et al., 2019).

Preprocessed data from the ADHD and TD samples were concatenated and entered into a group independent component analysis (ICA) to identify large-scale networks in the combined population (MELODIC; http://fsl.fmrib.ox.ac.uk/fsl/fslwiki/MELODIC). The number of components was set to 30, and four components (salience (SN), left and right frontoparietal (FPN), and default mode (DMN) networks) were identified using a quantitative template-matching procedure (Supekar et al., 2019, Greicius et al., 2004). The template matching procedure involved taking the average z score of voxels falling within the template minus the average z score of voxels outside the template and selecting the component in which this difference (the goodness of fit) was the greatest. The templates for SN, DMN, left FPN, and right FPN were identified from previously published studies (Uddin et al., 2011, Smith et al., 2009, Shirer et al., 2012, Miller et al., 2016). Three investigators (YM, WC, KS) then visually inspected the spatial maps and temporal profiles of each of the 30 ICA components and confirmed the selected SN, DMN, left FPN, and right FPN components (Supplemental Fig. S1).

### 2.5. Dynamic time-varying cross-network interactions

**Fig. 2.**
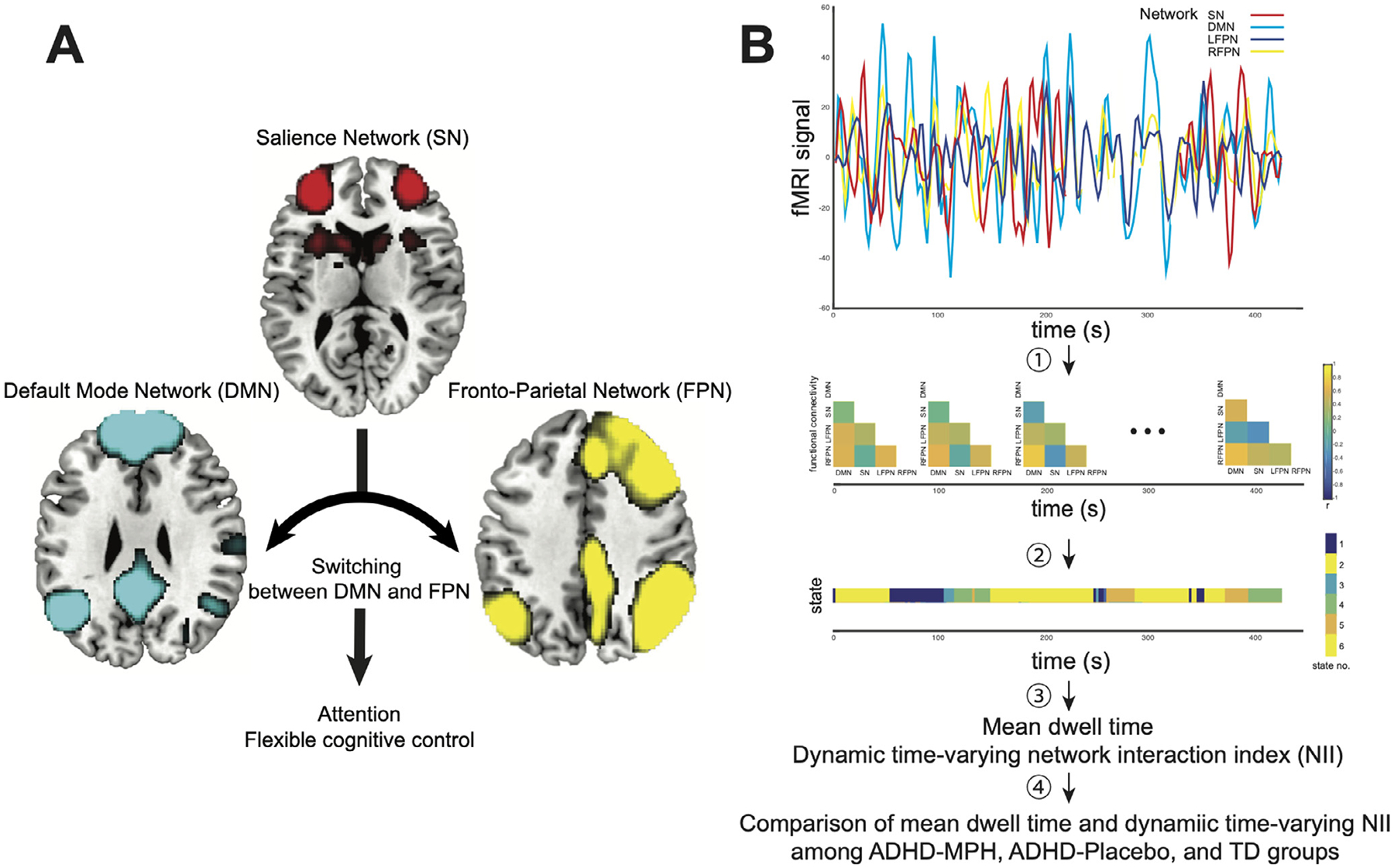
(A) Triple-network model of attention-deficit/hyperactivity disorder (ADHD). The model posits a key role for the salience network (SN) in altered dynamic temporal interactions with the frontoparietal network (FPN), and the default mode network (DMN), resulting in dysfunction of cognitive control. (B) Analysis pipeline for examining dynamic time-varying cross-network interactions within the triple-network model. Briefly, (1) We estimated dynamic functional interactions among the SN, FPN, and DMN using an exponentially decaying sliding window. (2) To identify distinct group-specific states associated with dynamic functional connectivity we applied group-wise k-means consensus-clustering on the time series of correlation matrices in each group separately. (3) We computed the mean dwell time and dynamic time-varying network interaction index (NII) for each brain state in each participant. (4) We examined differences in mean dwell time and dynamic time-varying NII across brain states among children with ADHD under osmotic release oral system methylphenidate administration (ADHD-MPH), children with ADHD under the placebo condition (ADHD-Placebo), and typically-developing (TD) groups. fMRI, functional magnetic resonance imaging; s, second.

Dynamic time-varying cross-network interactions among the SN, FPN, and DMN were measured using a dynamic functional connectivity approach (Cai et al., 2018, Zalesky et al., 2014, Allen et al., 2014). Our overall analysis pipeline is illustrated in Fig. 2 and described in detail in the Supplemental Methods. Briefly, we first estimated dynamic functional interactions among the SN, FPN, and DMN using an exponentially decaying sliding window. Second, we identified distinct group-specific states associated with dynamic functional connectivity using a group-wise *k*-means consensus-clustering approach (Charrad et al., 2014). The optimal number of clusters in each group was determined on the basis of the majority vote of 30 indices which characterize the number of clusters. Third, we computed the mean dwell time in each brain state for each participant based on the average time spent continuously in that state. Note that a small number of states is not necessarily accompanied with long dwell time, or vice-a-versa. For example, with two latent brain states, it is possible that these states switch rapidly and frequently, resulting in very short dwell times. Fourth, we characterized cross-network interaction in each dynamic brain state using a brain state-specific network interaction index (NII) based on the hypothesized role of the SN in switching interactions with the FPN and DMN (Menon and Uddin, 2010, Menon, 2015). NII has the advantage of capturing interactions simultaneously among all three networks. Specifically, NII was computed as the difference in correlation between the SN and FPN time series and correlation between the SN and DMN (Menon, 2015, Greicius et al., 2003). NII thus captures the extent to which the SN temporally engages with the FPN and dissociates itself from the DMN (Menon, 2015, Greicius et al., 2003).

$$NII=f\left(CC_{SN,FPN}\right)-f(CC_{SN,DMN})$$

where

$$f(CC)=\frac{1}{2}In\left(\frac{1+cc}{1-cc}\right)$$

CC is Pearson’s correlation between the time series of two component networks, e.g., CC_*SN*, *DMN*_ refers to the correlation between the time series of the SN and DMN. *f*(*CC*) computes Fisher z-transform of Pearson’s correlation (*CC*) between ROI timeseries. Thus for instance, *f*(*CC*_*SN,FPN*_) computes Fisher z-transform of the Pearson’s correlation between the time series of the SN and FPN. *f*(*CC*_*SN, L FPN*_) and *f*(*CC*_*SN, R FPN*_) were computed separately and then their average was used as *f* (*CC*_*SN,FPN*_). Larger NII values reflect more segregated cross-network interactions between the SN-FPN and SN-DMN systems in the context of the triple-network model.

We computed an NII for each sliding window and averaged NIIs for the windows corresponding to the same dynamic brain state. We next computed the mean and variability (measured by standard deviation) of time-varying NIIs across all the dynamic brain states for each participant.

### 2.6. Statistical analysis

First, we examined administration-related changes in sustained attention, as indexed by CPT performance measures, between the ADHD-MPH and ADHD-Placebo conditions using paired *t*-tests.

Second, we examined administration-related changes in mean dwell times and mean and variability of dynamic time-varying NII between the ADHD-MPH and ADHD-Placebo conditions using paired *t*-tests. Next, we examined differences by contrasting the ADHD-Placebo and TD control groups using two-sample *t*-tests. Finally, we examined administration-related improvement using two-sample *t*-tests contrasting the ADHD-MPH and TD control groups. A Bonferroni correction was used to correct for multiple comparisons across groups.

Third, to investigate the robustness of our findings in the context of methylphenidate-related improvement, which is the focus of the present study, we examined whether multivariate dynamic time-varying NII measures could distinguish ADHD-MPH, ADHD-Placebo, and TD control groups. We used a Regression Tree classifier (Breiman et al., 1984) and leave-one-out cross-validation with the mean and variability of time-varying NII as features to test the performance of the classifier. We chose NII over dwell time because it is based on the triple-network model which we sought to test. Results of additional analyses using dwell time features are reported in the Supplemental Materials. Data from one participant was selected as a test set and the rest of the data were used as a training set. The training set was then used to train a classification model, which was then applied to classify the test set. This process was repeated N times with each participant’s data used exactly once for testing. The significance of classification accuracy was evaluated using parametric test that determines whether the accuracy is better than the no information rate, which is taken to be the largest class percentage in the data. The aforementioned classification analysis was performed using the *caret* R package (https://cran.rproject.org/web/packages/caret/).

Fourth, to evaluate the behavioral relevance of dynamic time-varying cross-network interactions between the SN, FPN and DMN, we examined its relation with inattention and hyperactivity/impulsivity scores under no medication evaluated on the Conners ADHD scale. We evaluated this relation across the ADHD-Placebo and TD groups using *Pearson’s* correlation, similar to our previous study (Cai et al., 2018), as ADHD symptoms exist on a continuum (McLennan, 2016). Bonferroni corrections were used to correct for the number of clinical measures tested, for each measure of dynamic connectivity.

Finally, multiple linear regression analysis was conducted to examine whether dynamic time-varying NII (i.e. under placebo condition) could predict the effect of methylphenidate on sustained attention. The composite performance score in CPT was used as measures of sustained attention, and was set as dependent variables. Mean and standard deviation of dynamic time-varying NII under placebo were set as independent variables with age, FSIQ, mean FD as confounding factors.

Demographic data are expressed as the mean ± SD. The clinical values were compared using paired *t*-test (comparison between ADHD-MPH and ADHD-Placebo) and Welch’s *t*-test (comparison with TD controls) for numerical variables and chi-square tests for categorical valuables. All statistical tests (Welch’s *t*-test, chi-square test, and *Pearson’s* correlation coefficients) were parametric test and two-tailed; *p*-values less than 0.05 were considered statistically significant.

## 3. Results

### 3.1. Demographic and clinical characteristics

Data analyses involved data from 76 male subjects, comprising 27 patients with ADHD (age: 10.6 ± 1.8 years, range 7.3–15.5 years) and 49 TD controls (age: 11.1 ± 2.3 years, range 6.1–15.6 years) (Table 1). There were no significant differences in age, sex, and handedness between the ADHD and TD groups (all *ps* > 0.05). The two groups showed significant differences in inattention, hyperactivity/impulsivity and FSIQ (all *ps* < 0.001). Mean FD in the ADHD-MPH was significantly lower than the ADHD-Placebo and TD groups (*ps* < 0.001, = 0.002). There were no differences in mean FD between the ADHD-Placebo and TD groups (*p* = 0.450). Detailed information about study participants, including comorbidity and medication history, is provided in Table 1.

### 3.2. Effect of methylphenidate on sustained attention in children with ADHD

**Fig. 3.**
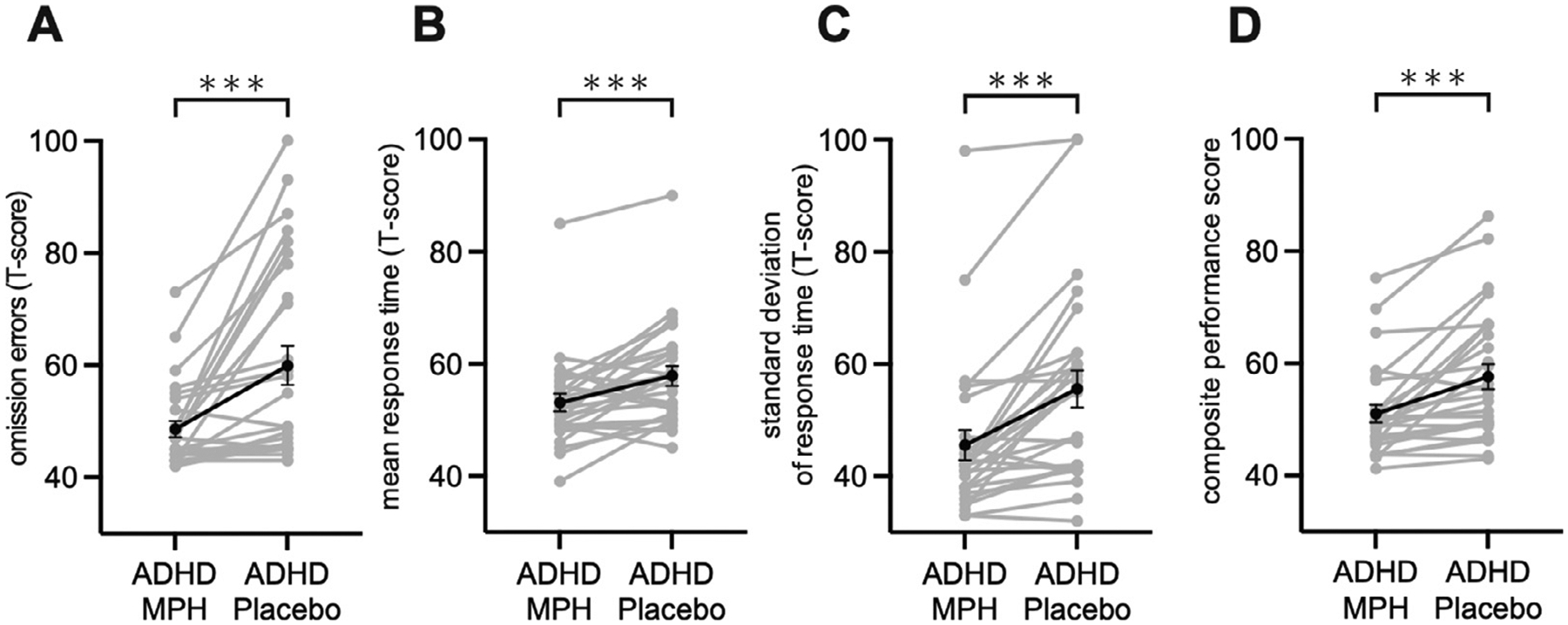
Sustained attention on continuous performance task in children with attention-deficit/hyperactivity disorder (ADHD) under osmotic release oral system methylphenidate administration (ADHD-MPH) and placebo condition (ADHD-Placebo). (A) Omission errors, (B) Mean response time, (C) Standard deviation of response time, (D) Composite performance score based on omission and commission errors, mean response time, and standard deviation of response time. Methylphenidate improved omission errors, mean response time, standard deviation of response time, and composite performance score. ***p < 0.001

To investigate the behavioral consequences of methylphenidate administration, we first examined sustained attention, assessed using the CPT in the ADHD-MPH and ADHD-Placebo conditions. Omission errors, mean RT, and standard deviation of RT were significantly lower in the ADHD-MPH, compared to the ADHD-Placebo, condition (all *ps* < 0.001, *ts* (26) = 4.03, 3.87, 5.05, Cohen’s *ds* = 0.78, 0.75, 0.97, respectively; Fig. 3 A, B, C). There was no significant difference in commission errors between the two conditions (*p* = 0.857, *t* (26) = 0.18, Cohen’s *d* = 0.03). We then examined composite scores, computed using all four behavioral measures, and found that it was significantly lower in the ADHD-MPH, compared to the ADHD-Placebo, condition (*p* < 0.001, *t* (26) = 4.60, Cohen’s *d* = 0.89; Fig. 3 D). These results suggest that a single dose of methylphenidate improves sustained attention deficits in children with ADHD.

### 3.3. Effect of methylphenidate on dynamic time-varying cross-network interactions in children with ADHD

**Fig. 4.**
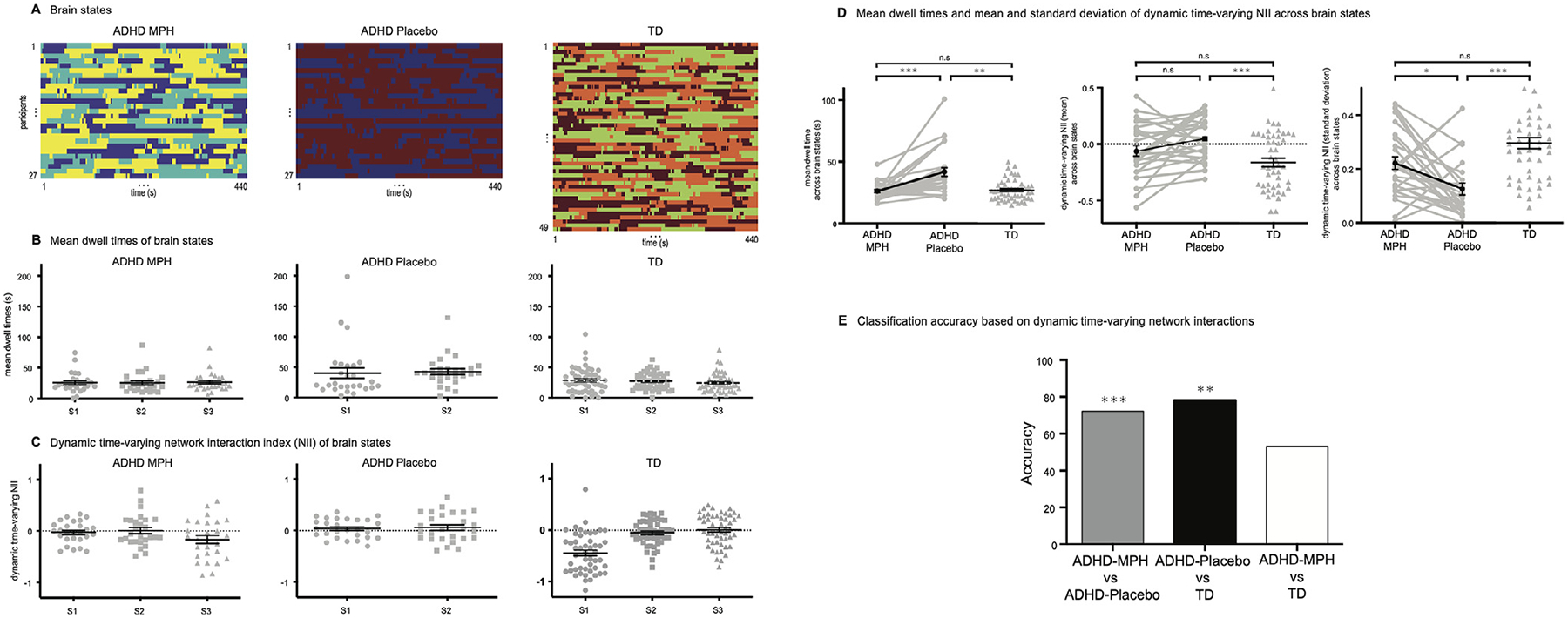
Dynamic time-varying cross-network interactions among cognitive control networks of brain states in children with attention-deficit/hyperactivity disorder (ADHD) under osmotic release oral system methylphenidate administration (ADHD-MPH) and placebo condition (ADHD-Placebo), and typically-developing (TD) group. (A) Dynamic time-varying brain states in children with ADHD-MPH and ADHD-Placebo, and TD group. Color indicates distinct states in each participant. (B) Mean dwell time of brain states. (C) Dynamic time-varying network interaction index (NII) of brain states (D) Mean dwell time, mean and variability of dynamic time-varying NII across brain states were aberrant in the ADHD-Placebo compared to the TD group. Methylphenidate remediated mean dwell time and variability of time-varying NII in children. (E) Classification analysis revealed that dynamic time-varying cross-network interactions can distinguish between the ADHD-MPH and ADHD-Placebo groups with an accuracy of 72%, and between the ADHD-Placebo and TD groups with an accuracy of 78%. In contrast, no differences were observed between the ADHD-MPH and TD groups (accuracy = 53%). ***p < 0.001; **p < 0.01; *p < 0.05; n.s, not significant.

To investigate the brain circuit mechanisms that underlie methylphenidate administration, we examined dynamic time-varying functional interactions between the SN, FPN and DMN. We first used temporal clustering analysis of time varying connectivity to identify distinct states in each group. This analysis revealed three states in the ADHD-MPH group, two states in the ADHD-Placebo group, and three states in the TD group (Fig. 4 A, B, Supplemental Fig. S6). We next compared mean dwell times across brain states among the ADHD-MPH, ADHD-Placebo and TD control groups. Mean dwell times were significantly shorter in the ADHD-MPH compared to the ADHD-Placebo condition (*p* < 0.001, Bonferroni corrected, *t* (26) = 4.37, Cohen’s *d* = 0.84). Compared to the TD group, mean dwell times were significantly longer in the ADHD-Placebo group (*p* = 0.001, Bonferroni corrected, *t* (32) = 3.94, Cohen’s *d* = 1.15), but was not significantly different in the ADHD-MPH group (*p* = 1, Bonferroni corrected, *t* (64) = 0.54, Cohen’s *d* = 0.12) (Fig. 4 D).

We next probed mean and variability of dynamic time-varying cross-network interactions among the three groups (Fig. 4 C).This analysis focused on SN-centered measures of network interaction index (NII) (Cai et al., 2018, Supekar et al., 2019). Mean of time-varying NII was not significantly different between the ADHD-MPH and the ADHD-Placebo conditions (*p* = 0.132, Bonferroni corrected, *t* (26) = 2.12, Cohen’s *d* = 0.41). Mean of time-varying NII was significantly higher in the ADHD-Placebo than the TD group (*p* < 0.001, Bonferroni corrected, *t* (66) = 3.94, Cohen’s *d* = 0.87), but was not significantly different between the ADHD-MPH and TD groups (*p* = 0.290, Bonferroni corrected, *t* (58) = 1.69, Cohen’s *d* = 0.40) (Fig. 4 D).

Variability of time-varying NII was significantly higher in the ADHD-MPH compared to the ADHD-Placebo condition (*p* = 0.019, Bonferroni corrected, *t* (26) = 2.97, Cohen’s *d* = 0.57). Compared to the TD group, NII variability was significantly lower in the ADHD-Placebo (*p* < 0.001, Bonferroni corrected, *t* (63) = 5.75, Cohen’s *d* = 1.30), but was not significantly different in the ADHD-MPH (*p* = 0.053, Bonferroni corrected, *t* (61) = 2.43, Cohen’s *d* = 0.56). Additional analyses confirmed that our main findings held even after controlling for comorbidity, FSIQ and mean FD as potential confounds (Supplemental Fig. S2, Supplemental Table S1, S2, S3). Supplemental analyses further revealed that static time-averaged NII measures yielded convergent findings, albeit with smaller effect sizes than variability of dynamic time-varying NII (Supplemental Fig. S3). These results indicate that methylphenidate improves mean dwell time of dynamic brain states, as well as variability of dynamic SN-centered network interactions in children with ADHD.

### 3.4. Classification analysis of effect of methylphenidate on dynamic time-varying cross-network interactions in children with ADHD

We examined whether dynamic time-varying cross-network interactions could distinguish among ADHD-MPH, ADHD-Placebo, and TD control groups, using a classifier with mean and variability of time-varying NII as features (Fig. 4 E). Dynamic time-varying cross-network interactions distinguished between the ADHD-MPH and ADHD-Placebo groups with an accuracy of 72% (*p* < 0.001), and between the ADHD-Placebo and TD groups with an accuracy of 78% (*p* = 0.009). In contrast, no differences were observed between the ADHD-MPH and TD groups (accuracy = 53%; *p* = 0.978). These results further demonstrate that methylphenidate improves dynamic connectivity patterns associated with the SN, FPN and DMN. Furthermore, we found dynamic time-varying cross-network interactions could distinguish ADHD vs TD groups from the two independent ADHD-200 cohorts used in our prior study (Cai et al., 2018) - an New York University (NYU) cohort and a Peking University (PKU) cohort (Supplemental Fig. S4).

### 3.5. Relation between dynamic time-varying cross-network interactions and ADHD clinical symptom measures

Next, we examined dynamic cross-network interactions and their relation to inattention and hyperactivity/impulsivity assessed by the Conners ADHD scale (Conners et al., 2008). Mean dwell time was correlated with inattention (*r* = 0.40, *p* < 0.001, Bonferroni corrected) and hyperactivity/impulsivity (*r* = 0.41, *p* < 0.001, Bonferroni corrected). Mean of time-varying NII was correlated with inattention (*r* = 0.30, *p* = 0.019, Bonferroni corrected) and hyperactivity/impulsivity (*r* = 0.24, *p* = 0.076, Bonferroni corrected). Variability of time-varying NII was correlated with inattention (*r* = −0.46, *p* < 0.001, Bonferroni corrected) and hyperactivity/impulsivity (*r* = −0.45, *p* < 0.001, Bonferroni corrected) (Supplemental Fig. S5). These results suggest that dynamic time-varying cross-network interactions among SN, FPN, and DMN are clinically relevant.

### 3.6. Brain dynamics-based predictors of the effect of methylphenidate on sustained attention

Finally, we used multiple linear regression analysis to determine whether mean and variability of dynamic time-varying NII, as well as age, FSIQ, and mean FD, under the placebo condition could predict individual differences in treatment response. Results showed that variability of time-varying NII in the ADHD-Placebo group predicted MPH-induced changes in composite CPT scores (*β* = −26.57, *p* = 0.043, Supplemental Table S4). These results suggest that dynamic time-varying cross-network interaction predict individual differences in treatment response.

## 4. Discussion

We investigated dynamic brain circuit mechanisms underlying the therapeutic effects of methylphenidate in childhood ADHD. We used a randomized placebo-controlled double-blind crossover design and a systems neuroscience-based model focusing on the SN, FPN and DMN, three core brain networks involved in cognitive control and implicated in ADHD. Methylphenidate improved sustained attention, and remediated aberrancies in brain network dynamics, including dwell time of latent brain states and time-varying cross-network interactions, among the SN, FPN and DMN. These findings were robust against potential confounds including comorbidity, IQ, and head motion. Importantly, dynamic time-varying network interactions under the placebo condition predicted methylphenidate-induced improvements in sustained attention. Our findings demonstrate that a single dose of methylphenidate can remediate aberrant brain dynamics among three core cognitive control networks implicated in ADHD. More generally, our findings identify a brain circuit mechanism underlying response to pharmacological treatment of childhood ADHD and identify a potential biomarker for predicting treatment response.

### 4.1. Methylphenidate improves sustained attention in children with ADHD

Our first goal was to determine whether methylphenidate administration improves cognitive performance on a sustained attention continuous performance task (Huang-Pollock et al., 2012, Shin et al., 2000). A single dose of methylphenidate lowered omission errors, mean RT, and standard deviation of RT reflecting improved performance (Huang-Pollock et al., 2012, Park et al., 2011, Wang et al., 2011) and analysis of a composite performance measure confirmed our finding. Specifically, results suggest that methylphenidate improves inattention, as measured by omission errors, and information processing speed, as measured by mean RT, and consistency as measured by standard deviation of RT over trials (Fujioka et al., 2016, Shin et al., 2008). Our findings that methylphenidate improves sustained attention are consistent with recent meta-analyses (Tamminga et al., 2016, Coghill et al., 2014), and add further evidence that a single dose of methylphenidate can be effective in ameliorating inattention and cognitive control deficits in children with ADHD.

### 4.2. Methylphenidate remediates aberrant functional brain network dynamics in children with ADHD

The next important goal of our study was to determine whether methylphenidate remediates aberrancies in functional networks associated with cognitive control in children with ADHD. Critically, this is the first study to examine the effect of methylphenidate on brain network dynamics in children with ADHD and with sample sizes greater than extant related randomized controlled studies (Silk et al., 2017, An et al., 2013).

We identified two features associated with aberrant functional brain network dynamics in children with ADHD under the placebo condition. First, compared to TD controls, children with ADHD showed significant differences in latent brain states associated with interaction among the three networks. Latent brain states were determined using temporal clustering, such that each state was characterized by a distinct pattern of functional interactions among the SN, FPN, and DMN. This analysis revealed that children with ADHD in the placebo condition had longer dwell times in individual brain states than TD controls. Methylphenidate decreased dwell times in children with ADHD, when compared to the placebo condition. Furthermore, following treatment, children with ADHD were no longer distinguishable from TD controls in their dwell times across brain states.

Second, we further characterized cross-network interactions in each brain state using a brain state-specific network interaction index (NII). The NII is a parsimonious metric based on the triple-network model (Menon and Uddin, 2010, Menon, 2015), which suggests that the SN plays a critical role in allocating cognitive resource by switching its interaction with the FPN and DMN. The SN and FPN are co-activated during high demanding cognitive task whereas the DMN is decoupled from the SN and FPN and is anti-correlated with both the SN and FPN (Cai et al., 2018, Supekar et al., 2019). Thus, the NII metric captures differences in dynamic engagement between the SN and FPN and dis-engagement between the SN and DMN. Analysis of time-varying NIIs across dynamic brain states, computed in each participant, revealed that under the placebo condition, children with ADHD showed significant differences in variability of interaction among the three networks compared to TD controls. Methylphenidate significantly changed variability of time-varying NIIs in children with ADHD, when compared to the placebo condition. Furthermore, following treatment, children with ADHD were more similar to TD controls in their SN-related network dynamics. Classification analyses further supported the finding that methylphenidate remediates aberrant dynamic brain network interactions.

Our findings suggest that a single dose of methylphenidate can remediate aberrant dynamic brain network interactions among the SN, FPN and DMN. Furthermore, we found the relation between dynamic cross-network interactions and ADHD symptoms. These results suggests the behavioral relevance of dynamic cross-network interactions between the SN, FPN and DMN in the present sample, consistent with a previous report in childhood ADHD (Cai et al., 2018), and broadly consistent with the role of triple-network interactions in flexible allocation of attention and cognitive control resources (Cai et al., 2016, Chen et al., 2015, Supekar and Menon, 2012, Sridharan et al., 2008, Menon and D’Esposito, 2022).

### 4.3. Integrative perspective on dopamine and modulation of cognitive control systems in children with ADHD

Our findings inform theoretical models of cognitive control circuits in childhood ADHD and their alteration by dopaminergic medication. The triple-network model proposes that the SN facilitates access to attention and cognitive control resources and plays a crucial role in switching its interaction with the FPN and DMN involved in externally-oriented attention and internally-oriented mental processes (Menon and Uddin, 2010). Prominent activation in SN and its interaction with FPN and DMN have been well documented in cognitive control tasks, such as the continued performance and stop-signal tasks (Cai et al., 2016, Cai et al., 2019). Altered interactions between these networks have been linked to cognitive control and attentional deficits in neurotypical individuals as well as children with ADHD (Cai et al., 2018, Braun et al., 2015, Chen et al., 2016). Our findings suggest that restoring dynamic network interactions between the SN, FPN and DMN may be one mechanism by which methylphenidate administration remediates deficits in sustained attention in children with ADHD. Methylphenidate acts on the dopamine transporter to inhibit reuptake of dopamine into synapses thereby increasing dopamine availability (Sulzer et al., 2005). Patients with ADHD, in comparison to healthy controls, have higher dopamine transporter density and increased dopamine transporter binding, resulting in low levels of dopamine (Sulzer et al., 2005) Critically, in neurotypical adults, mesolimbic dopamine capacity assessed using positron emission tomography (PET), has been linked to functional integrity of the SN (McCutcheon et al., 2019). Taken together, these observations suggest that amplification of dopamine signaling is a likely mechanism by which methylphenidate remediates aberrancies in SN-centered dynamics of cognitive control circuits in childhood ADHD.

### 4.4. Limitations and future work

Our study examined the effects of a single dose of OROS-MPH administration on sustained attention and dynamic brain circuits involved in cognitive control. Immediate release methylphenidate is not approved for clinical use in Japan. Critically, methylphenidate is the main active ingredient in both osmotic and immediate release medications. In our study, the resting-state fMRI was acquired 5–8 hours after medication when methylphenidate concentrations in the blood generally reach their maximum (American Psychiatric Association 2007), thus, matching immediate release methylphenidate as closely as possible. However, no studies have directly compared brain response to osmotic release versus immediate-release methylphenidate, so the precise correspondence remains to be investigated.

While our design allowed us investigate mechanisms by which cognitive control is improved by methylphenidate, it did not include follow up clinical assessments, so the overall real-life longitudinal clinical effects are not known. Further studies with multi-dose methylphenidate administration, treatment duration and stimulant formulations, and follow up assessments of inattention, hyperactivity, and impulsivity are needed to determine the long-term clinical impact of our findings (Pereira-Sanchez et al., 2021, Pereira-Sanchez et al., 2021).

In the present study, we found that dynamic time-varying NII between the SN, FPN and DMN was lower in children with ADHD under placebo condition compared to TD controls at baseline, an opposite pattern of those reported in a previous study (Cai et al., 2018). This discrepancy could be in part due to differences in scanning protocols, participant selection criteria, sex ratio, and medication history, which could influence assessments of brain states across studies, as highlighted by recent comprehensive reviews of fMRI studies of ADHD (Cortese et al., 2021, Pereira-Sanchez and Castellanos, 2021). Given the considerable heterogeneity that characterizes ADHD both at the behavioral phenotypic level and the underlying intrinsic functional connectivity measures level (Castellanos and Aoki, 2016), further work with larger samples is needed to characterize heterogenous profiles of dynamic brain circuit patterns across different ADHD cohorts.

Our NII metric focuses on three core cognitive control systems involving the SN, FPN and DMN. Missing here are interactions basal ganglia and reward pathways implicated in ADHD, which need to be incorporated into future work. As with most studies of ADHD, children with ADHD in our study were not drug naïve, were male, and spanned a wide range from 5 to 16. Specifically, all participants were medication-free prior to MRI for at least 5 half-lives. For ethical reasons we could not stop children’s medication between study days. Our randomized placebo-controlled double-blind crossover design involved similar washout protocols for the methylphenidate and placebo groups, thus controlling for the effects of use of medication between study days. The effect of medication on cognition and brain activity are likely to be influenced by administration procedures, the length of washout and medication dosage. Larger studies that include drug naïve males and females with ADHD are needed to determine how medication history, sex, and development stage along with other medication factors modulate methylphenidate effects on aberrant cognitive control circuits (Mueller et al., 2014). For ethical reasons, while we used a randomized controlled design for children with ADHD, TD controls were only studied at baseline. Designs that incorporate methylphenidate and placebo arms in TD controls, with multiple measures of behavioral and clinical measures associated with ADHD, may provide further insights into how methylphenidate impacts cognitive control circuits and sustained attention.

### 4.5. Conclusions

Our randomized placebo-controlled double-blind crossover study demonstrates, for the first time, that a single dose of methylphenidate can improve sustained attention and remediate aberrant brain circuit dynamics in cognitive control circuits in children with ADHD. Our findings provide novel insights into brain mechanisms underlying successful methylphenidate administration in ADHD and may lead to clinically useful biomarkers for evaluating ADHD treatment. More generally, salience network-related cross-network dynamics provides a novel and parsimonious quantitative systems-neuroscience based template for investigating the neural consequences of therapies that treat cognitive and attention deficits in psychiatric and neurological disorders. Importantly, our study addresses fundamental gaps in our knowledge of dopaminergic action on cognitive control circuits in children with ADHD.

## Supplementary Material

1

## Figures and Tables

**Table 1:** Demographic and behavioral characteristics of participants included in data analysis.

	ADHD		TD	Statistic	*p*
	MPH	Placebo			
**Sample size**	27		49	-	-
**Age (years)**	10.7±1.8		11.1±2.3	*t* (66) = 0.85	0.397
**Handedness (R/L)**	25/2		47/2	*χ*^2^ (1) = 0.39	0.534
**FSIQ**	90.8±8.7		105.2±11.0	*t* (65) = 6.27	<0.001***
**Conners IN (T)**	78.4±12.1		45.7±8.5	*t* (41) = 12.48	<0.001***
**Conners HY (T)**	73.1±15.3		42.9±3.9	*t* (28) = 10.07	<0.001***
**mean FD (mm)**	0.058±0.014	0.082±0.041	0.075±0.033	*t* (26) = 3.60	<0.001*** (MPH vs Placebo)
				*t* (70) = 3.18	0.002** (MPH vs TD)
				*t* (45) = 0.76	0.450 (Placebo vs TD)
**Medication use**	medication-naïve 1		-	-	-
	OROS-MPH 25				
	atomoxetine 3				
	aripiprazole 2				
**Comorbidity**	none 14		-	-	-
	autism spectrum disorder 9				
	oppositional defiant disorder 6				
	specific learning disorder 2				
	developmental coordination disorder 1				

ADHD, attention-deficit/hyperactivity disorder; OROS-MPH, osmotic release oral system methylphenidate; TD, typically-developing; R, right; L, left; FSIQ, full scale intelligence quotient; IN, inattention; HY, hyperactivity/impulsivity; FD, framewise displacement;

*** *p* < 0.001,

** *p* < 0.01.

## Data Availability

Data and code may be provided to interested researchers upon reasonable request to the corresponding author, after clearance from the Research Ethics Committee.

## Abbreviations:

ADHD: attention deficit/hyperactivity disorder
TD: typically-developing
MPH: methylphenidate
OROS-MPH: osmotic release oral system methylphenidate
SN: salience network
DMN: default mode network
FPN: frontoparietal network
NII: network interaction index
CPT: continuous performance task
