# Supplementary material for: Methylphenidate remediates aberrant brain network dynamics in children with attention-deficit/hyperactivity disorder: A randomized controlled trial
Source: Neuroimage. Author manuscript; Available in PMC 2022 Aug 15. (PMC9286726; doi:10.1016/j.neuroimage.2022.119332)
Supplement: 1 [file NIHMS1819024-supplement-1.docx]

**Supplemental Materials**

1. Supplemental Methods 2

1.1 Study design and participants 2

1.2 Dynamic time-varying cross-network interactions 5

1.3 Similarity between latent brain states 7

2. Supplemental Results 8

2.1 Mean dwell time and dynamic time-varying NII across brain states controlling for FSIQ and movement 8

2.2 Mean dwell time and dynamic time-varying NII across brain states excluding children with comorbid disorders 9

2.3 Static time-averaged cross-network interactions 10

2.4 Classification based on dynamic time-varying cross-network interactions in an New York University (NYU) cohort and a Peking University (PKU) cohort. 11

2.5 Functional connectivity patterns per state in ADHD-MPH, ADHD-Placebo, and TD controls group 12

3. Supplemental Figures 13

Supplemental Figure S1 13

Supplemental Figure S2 14

Supplemental Figure S3 15

Supplemental Figure S4 16

Supplemental Figure S5 17

Supplemental Figure S6 19

4. Supplemental Tables 20

Supplemental Table S1 20

Supplemental Table S2 21

Supplemental Table S3 22

Supplemental Table S4 23

Supplemental Table S5 24

5. Supplemental References 25

**1. Supplemental Methods**

**1.1 Study design and participants**

The overall design of the study is shown in **Figure 1**. 34 children with attention-deficit/hyperactivity disorder (ADHD) were recruited at the University of Fukui Hospital, Japan. Recruitment started in 24/6/2017, and ended in 21/4/2018 because the number of enrolled cases reached the target number. The diagnosis of ADHD was based on the Diagnostic and Statistical Manual of Mental Disorders, Fifth Edition (DSM-5)^1^, and was confirmed in structured interviews with investigators using the ADHD module of the Japanese Version of the Kiddie Schedule for Affective Disorders and Schizophrenia for School-Aged Children-Present and Lifetime Version (K-SADS-PL-J)^2,3^. Comorbid conditions were evaluated using a semi-structured diagnostic interview, via the Mini International Neuropsychiatric Interview for Children and Adolescents - Japanese version (MINI KID)^4^. Additionally, 65 typically-developing (TD) children were recruited as control participants from the community between 13/6/2015 and 8/12/2018. The presence of no psychiatric diseases in TD controls was confirmed by MINI KID^4^. The TD control group had no family history of psychiatric diseases. Intellectual capacities were estimated via the Wechsler Intelligence Scale for Children-Fourth (WISC-IV)^5^. Parents of children in both groups were asked to complete Conners 3rd Edition (Conners) instrument^6^ to evaluate inattention, and hyperactivity/impulsivity symptoms under no medication. Handedness was assessed using the Edinburgh Handedness Inventory^7^.

Inclusion criteria for both groups were no contraindications for magnetic resonance imaging (MRI), full scale intelligence quotient (FSIQ) > 70, no history of severe head trauma or neurological abnormalities (e.g. epilepsy, arachnoid cysts). To minimize the potential impact of sex differences we included only male participants, consistent with the male bias in the prevalence of ADHD^8,9^. Participants with excessive head motion (over 3.0 mm, 3.0 degree, and mean framewise displacement (FD) 0.3 mm) during the scanning were excluded^10–12^.

33 children with ADHD were first randomly assigned to methylphenidate or placebo conditions. One child with ADHD declined to participate, and was therefore excluded. The study drug manager assigned the study drug to participants using block randomization. Each time a participant was enrolled, the study drug was assigned according to label of the study drug which was recorded in a random order in advance. During the first visit, participants either took a single dose of osmotic release oral system methylphenidate (OROS-MPH; 1.0mg/kg: 1.0 ± 0.1mg/kg)^13^ or a placebo (lactose) under double-blind conditions, as previous studies^13,14^. We chose OROS-MPH because immediate release prescriptions are prohibited in Japan. Five to eight hours after administration, when methylphenidate concentration in the blood is maximal^15^, participants underwent a resting-state functional MRI (fMRI) scan. Children with ADHD were also administered a continuous performance task (CPT)^16^ outside the MRI scanner. Of the 17 children with ADHD who received OROS-MPH, 15 completed both MRI and CPT, and 2 were excluded because they did not stay still during the MRI scan. Of 16 children with ADHD who received the placebo, 15 completed MRI and CPT, and 1 was excluded because an arachnoid cyst was detected.

At the second visit, within 1 to 6 weeks from the first visit (mean 17.9 days, standard deviation 9.6 days), they underwent a second resting-state fMRI and performed the CPT again following administration of either the placebo or OROS-MPH under double-blind conditions. Children with ADHD who took a single dose of OROS-MPH at the first visit now took the placebo at the second visit, and vice versa. All 15 children with ADHD who received OROS-MPH completed MRI and CPT, and of the 15 children with ADHD who received the placebo, 12 completed MRI and CPT, and 3 were excluded because 1 declined MRI, and 2 did not stay still during the MRI scan. Adverse events on OROS-MPH condition were 2 loss of appetite, 2 insomnia, 1 abdominal pain, and 1 fatigue, all of which were temporal and mild. No serious adverse events were observed. Of 65 TD children, 16 were excluded, as 6 were female, 6 had psychiatric disorders, and 4 had neurological abnormalities. TD controls were scanned once without OROS-MPH or placebo.

Data analyses involved data from 76 subjects, comprising 27 patients with ADHD and 49 TD controls. 14 children with ADHD were classified as combined presentation, and 13 were predominantly inattentive presentation. 9 patients with ADHD had autism spectrum disorder, 6 ADHD patients had oppositional defiant disorder, 2 had specific learning disorder, and 1 had developmental coordination disorder as comorbid disorders. While one of the patients with ADHD was medication-naïve, 25 were medicated with OROS-MPH, three with atomoxetine, and two with aripiprazole. All participants were medication-free prior to MRI for at least 5 times half-lives, including methylphenidate and atomoxetine, consistent with protocol from previous studies^12,17^.

**1.2 Dynamic time-varying cross-network interactions**

Dynamic time-varying cross-network interactions were measured using a dynamic functional connectivity approach, similar to our previous studies^18–20^. We estimated dynamic functional interactions between the SN, FPN, and DMN using an exponentially decaying sliding window with a window length of 18 TRs (41.4 seconds) and a sliding step of 1 TR (2.3 seconds)^18,20^. Exponentially decaying weights were applied to each time point within a window as described in previous studies^18,19^. Within each time window, we computed the z-transformed *Pearson's* correlation between the ICA time-series taken pairwise. This resulted in a time-series of correlation matrices (T x C), where T is the number of time windows and C is the number of pairwise interactions among SN, FPN, and DMN at each time point.

To identify distinct group-specific states associated with dynamic functional connectivity, we applied a group-wise *k*-mean consensus-clustering on the time-series of correlation matrices in each group separately with the number of clusters (k) ranging from 2 to 20. The optimal number of clusters in each group was determined on the basis of the majority vote of 30 indices of internal validity measures (NbClust package in R)^21^. Because our goal was to investigate whether dynamic temporal properties, including the number of brain states and their mean dwell times, differed among the ADHD group under OROS-MPH administration condition (ADHD-MPH), ADHD group under placebo condition (ADHD-Placebo), and TD groups, we allowed the number of clusters to differ among the three groups, rather than keeping them exactly the same^22^. To quantify dwelling time of dynamic brain states, we computed mean dwell time of each brain state for each participant, based on the average time spent continuously in that state.

Brain state-specific network interaction index (NII) was used to characterize cross-network interaction in each dynamic brain state. NII measures cross-network interactions among the three networks based on the hypothesized role of the SN in switching interactions with the FPN and DMN^23,24^. NII has the advantage of capturing interactions simultaneously among all three networks. Specifically, NII was computed as the difference in correlation between SN and FPN time series and the correlation between SN and DMN. NII thus captures the extent to which the SN temporally engages with the FPN, and dissociates itself from the DMN.

*NII = f* (*CC_SN,FPN_*) - *f* (*CC_SN,DMN_*)

where

*f* (*CC*) = $\frac{1}{２}ln(\frac{1+CC}{1-CC})$

CC is *Pearson’s* correlation between the time series of two component networks, e.g., CC*_SN_,_DMN_* refers to the correlation between the time series of the SN and DMN. 𝑓(𝐶𝐶) computes Fisher z-transform of *Pearson's* correlation (𝐶𝐶) between ROI timeseries. Thus for instance, (*CC_SN,FPN_*) computes Fisher z-transform of the *Pearson's* correlation between the time series of the SN and FPN. (*CC_SN,LFPN_*) and 𝑓(*CC_SN,RFPN_*) were computed separately and then their average was used as 𝑓(*CC_SN,FPN_*). Larger NII values reflect more segregated cross-network interactions between the SN-FPN and SN-DMN systems in the context of the triple-network model.

We computed NII for each sliding window and averaged NIIs for the windows corresponding to the same dynamic brain state. We next computed the mean and variability (measured by standard deviation) of time-varying NIIs across all the dynamic brain states for each participant.

In addition to dynamic NII measures, we computed static time-averaged NII (measured across the entire time series) as well.

**1.3 Similarity between latent brain states**

We used a Euclidean distance metric to quantify the dissimilarity of functional connectivity patterns between groups. The Euclidean distance (*d*) was defined as the square root of the sum of squares of the difference in functional connectivity weights between two states. The smaller the distance, the more similar the brain states. **2. Supplemental Results**

**2.1 Mean dwell time and dynamic time-varying NII across brain states controlling for FSIQ and movement**

The ADHD and TD groups showed significant differences in FSIQ (*p* < 0.001).

Mean FD in the ADHD-MPH (0.058±0.014mm) was significantly lower than the ADHD-Placebo and TD groups (*ps* < 0.001, = 0.002). There were no differences in mean FD between the ADHD-Placebo (0.082±0.041mm) and TD groups (0.075±0.033mm) (*p* = 0.450). Then, we conducted several additional analyses to rule out these potential confounding effects.

Mean dwell times were compared including FSIQ and mean FD as covariates in the model to control for potential confounding effects. Mean dwell times were significantly shorter in the ADHD-MPH compared to the ADHD-Placebo condition (*p* < 0.001, Bonferroni corrected). Compared to the TD control group, mean dwell times were significantly longer in the ADHD-Placebo (*p* = 0.006, Bonferroni corrected), but was not significantly different in the ADHD-MPH (*p* = 1, Bonferroni corrected) **(Supplemental Table S1**).

Mean of dynamic time-varying NII was compared including FSIQ and mean FD as covariates in the model to control for potential confounding effects. Mean of dynamic time-varying NII was not significantly different between the ADHD-MPH and ADHD-Placebo conditions (*p* = 0.630, Bonferroni corrected). Compared to the TD control group, mean of dynamic time-varying NII was significantly higher in the ADHD-Placebo (*p* < 0.001, Bonferroni corrected), but was not significantly different in the ADHD-MPH (*p* = 0.255, Bonferroni corrected) **(Supplemental Table S2**).

NII variability was compared including FSIQ and mean FD as covariates in the model to control for potential confounding effects. NII variability was significantly higher in the ADHD-MPH compared to the ADHD-Placebo condition (*p* = 0.012, Bonferroni corrected). Compared to the TD control group, NII variability was significantly lower in the ADHD-Placebo (*p* < 0.001, Bonferroni corrected), but was not significantly different in the ADHD-MPH (*p* = 0.435, Bonferroni corrected) **(Supplemental Table S3**).

**2.2 Mean dwell time and dynamic time-varying NII across brain states excluding children with comorbid disorders**

We compared mean dwell times and time-varying NII across brain states among ADHD-MPH (n = 14), ADHD-Placebo, and TD groups using children with ADHD without comorbid disorders to exclude the effect of comorbidity.

Mean dwell times were significantly shorter in the ADHD-MPH compared to the ADHD-Placebo condition (*p* = 0.048, Bonferroni corrected, *t* (13) = 2.77, Cohen’s *d* = 0.74). Compared to the TD control group, mean dwell times were not significantly different in the ADHD-Placebo (*p* = 0.068, Bonferroni corrected, *t* (14) = 2.56, Cohen’s *d* = 1.22), and the ADHD-MPH (*p* = 0.650, Bonferroni corrected, *t* (41) = 1.26, Cohen’s *d* = 0.27) **(Supplemental Figure S2**).

Mean of dynamic time-varying NII was not significantly different between the ADHD-MPH and ADHD-Placebo conditions (*p* = 0.631, Bonferroni corrected, *t* (13) = 1.32, Cohen’s *d* = 0.35). Compared to the TD control group, mean of dynamic time-varying NII was significantly higher in the ADHD-Placebo (*p* = 0.003, Bonferroni corrected, *t* (26) = 3.68, Cohen’s *d* = 0.97), but was not significantly different in the ADHD-MPH (*p* = 0.189, Bonferroni corrected, *t* (23) = 1.96, Cohen’s *d* = 0.56) **(Supplemental Figure S2**).

Variability of dynamic time-varying NII was significantly higher in the ADHD-MPH compared to the ADHD-Placebo condition (*p* = 0.017, Bonferroni corrected, *t* (13) = 3.31, Cohen’s *d* = 0.88). Compared to the TD control group, NII variability was significantly lower in the ADHD-Placebo (*p* < 0.001, Bonferroni corrected, *t* (42) = 7.41, Cohen’s *d* = 1.61), but was not significantly different in the ADHD-MPH (*p* = 0.458, Bonferroni corrected, *t* (21) = 1.49, Cohen’s *d* = 0.46) (**Supplemental Figure S2**).

**2.3 Static time-averaged cross-network interactions**

In addition to dynamic NII measures, we also computed static time-averaged NIIs (measured across the entire time series), and compared them among ADHD-MPH, ADHD-Placebo, and TD groups. Static time-averaged NII was not significantly different between the ADHD-MPH and the ADHD-Placebo conditions (*p* = 0.359, Bonferroni corrected, *t* (13) = 1.67, Cohen’s *d* = 0.43). Static time-averaged NII was significantly higher in the ADHD-Placebo than the TD group (*p* = 0.012, Bonferroni corrected, *t* (28) = 3.15, Cohen’s *d* = 0.83), but static time-averaged NII was not significantly different between the ADHD-MPH and TD groups (*p* = 0.655, Bonferroni corrected, *t* (25) = 1.26, Cohen’s *d* = 0.31).

The effect sizes of static time-averaged NII between the ADHD under placebo and TD controls were smaller than the effect size of variability of dynamic time-varying NII. These results are in line with a previous study of children with ADHD and TD controls, which also reported larger effect sizes in variability of dynamic time-varying, compared to static time-averaged, NII measures^25^. These findings suggest that, compared with static connectivity measures, dynamic connectivity measures are more useful not only for distinguishing children with ADHD from controls, but also for monitoring treatment effects in affected children.

**2.4 Classification based on dynamic time-varying cross-network interactions in an New York University (NYU) cohort and a Peking University (PKU) cohort.**

We examined whether the dynamic time-varying cross-network interactions could distinguish ADHD vs TD groups from the two independent ADHD-200 cohorts used in our prior study^25^ - an New York University (NYU) cohort and a Peking University (PKU) cohort. We found that dynamic time-varying cross-network interactions distinguished between ADHD and TD group with an accuracy of 78% (*p* < 0.001) in the NYU cohort and 82% (*p* < 0.001) in the PKU cohort (**Supplemental Figure S4**). These results demonstrate that multivariate features of dynamic time-varying cross-network interactions associated with the SN, FPN and DMN across cohorts reliably distinguish between ADHD and TD groups across cohorts.

Further, we also included mean dwell time in classification analysis in children with ADHD. As a result, accuracy was 83% (ADHD-MPH and ADHD-Placebo, *p* < 0.001), and 68% (ADHD-Placebo and TD, *p* = 0.277), and 57% (ADHD-MPH and TD, *p* = 0.939).

**2.5 Functional connectivity patterns per state in ADHD-MPH, ADHD-Placebo, and TD controls groups**

We showed the features of each dynamic brain state in order to be able to appreciate the data structure. **(Supplemental Figure S6)**. Each dynamic brain state is defined by unique functional connectivity between the SN, FPN and DMN. Three brain states were uncovered in TD, two in Placebo and three in MPH groups. S1 in MPH and S3 in TD, S2 in MPH and S2 in TD, and S3 in MPH and S1 in TD look corresponded and similar. To further confirm it quantitatively, we used a Euclidean distance metric. We hypothesized that the distance between dynamic brain states in ADHD-MPH and TD would be smaller than the distance between dynamic brain states in ADHD-Placebo and TD. Consistent with this hypothesis, we found that S3 in the ADHD-MPH group had the smallest distance with S1 in the TD, S2 in the ADHD-MPH had the smallest distance with S2 in the TD, and S1 in the ADHD-MPH had the smallest distance with S3 in the TD (**Supplemental Table S5**). These findings demonstrate that medication reduces dissimilarity in dynamic brain states between children with ADHD and TD children.

**3. Supplemental Figures**

**Supplemental Figure S1. Salience (SN), frontoparietal (FPN), and default mode (DMN) networks**. **(A)** SN, **(B)** left FPN (LFPN), **(C)** right FPN (RFPN), and **(D)** DMN. Group-level independent component analysis was used to identify these networks. Maps are displayed with threshold z-scores > 2.3.

**Supplemental Figure S2. Dynamic time-varying cross-network interactions excluding children with comorbid disorders. (A)** Mean dwell time (B) Mean of dynamic time-varying network interaction index (NII) (**C**) Variability of dynamic time-varying NII. ADHD-MPH, ADHD patients under osmotic release oral system methylphenidate administration condition; ADHD-Placebo, ADHD patients under placebo condition; TD, typically-developing; ****p* < 0.001; ***p* < 0.01; **p* < 0.05; n.s, not significant.

**Supplemental Figure S3. Static time-averaged network interactions in the ADHD-MPH and ADHD-Placebo, and TD children.** NII, network interaction index; ADHD-MPH, ADHD patients under osmotic release oral system methylphenidate administration condition; ADHD-Placebo, ADHD patients under placebo condition; TD, typically-developing; ***p* < 0.01; n.s, not significant.

**Supplemental Figure S4. Classification based on dynamic time-varying cross-network interactions in an New York University (NYU) cohort and a Peking University (PKU) cohort.** TD, typically-developing; ****p* < 0.001.

**Supplemental Figure S5. Relation between dynamic time-varying cross-network interactions and clinical symptoms. (A)** Relation between inattention severity and mean dwell time (*r* = 0.40, *p* < 0.001, Bonferroni corrected) **(B)** Relation between inattention severity and mean of dynamic time-varying network interaction index (NII) (*r* = 0.30, *p* = 0.019, Bonferroni corrected) **(C)** Relation between inattention severity and standard deviation of dynamic time-varying NII (*r* = -0.46, *p* < 0.001, Bonferroni corrected) **(D)** Relation between hyperactivity/impulsivity severity and mean dwell time (*r* = 0.41, *p* < 0.001, Bonferroni corrected) **(E)** Relation between hyperactivity/impulsivity severity and mean of dynamic time-varying NII (*r* = 0.24, *p* = 0.076, Bonferroni corrected) **(F)** Relation between hyperactivity/impulsivity severity and standard deviation of dynamic time-varying NII (*r* = -0.45, *p* < 0.001, Bonferroni corrected).

**Supplemental Figure S6. Functional connectivity patterns per state in ADHD-MPH, ADHD-Placebo, and TD control groups.** ADHD-MPH, ADHD patients under osmotic release oral system methylphenidate administration condition; ADHD-Placebo, ADHD patients under placebo condition; TD, typically-developing; DMN, default mode network; SN, salience network; LFPN, left frontoparietal network; RFPN, right frontoparietal network S, state.

**4. Supplemental Tables**

**Supplemental Table S1. Mean dwell time across brain states after controlling for FSIQ and movement.**

|  |  | *t* | *p* |
| --- | --- | --- | --- |
| ADHD-MPH vs ADHD-Placebo | group | 4.121 | <0.001*** |
|  | FSIQ | - | - |
|  | mean FD | 0.319 | 1 |
| ADHD-Placebo vs TD controls | group | 3.213 | 0.006** |
|  | FSIQ | 1.097 | 0.828 |
|  | mean FD | 0.336 | 1 |
| ADHD-MPH vs TD controls | group | 0.681 | 1 |
|  | FSIQ | 1.149 | 0.763 |
|  | mean FD | 1.532 | 0.390 |

ADHD, attention-deficit/hyperactivity disorder; ADHD-MPH, ADHD patients under osmotic release oral system methylphenidate administration condition; ADHD-Placebo, ADHD patients under placebo condition; FD, framewise displacement; FSIQ, full scale intelligence quotient; NII, network interaction index; TD, typically-developing; ****p* < 0.001; ***p* < 0.01, **p* < 0.05.

**Supplemental Table S2. Mean of dynamic time-varying NII across brain states after controlling for FSIQ and movement.**

|  |  | *t* | *p* |
| --- | --- | --- | --- |
| ADHD-MPH vs ADHD-Placebo | group | 1.28 | 0.630 |
|  | FSIQ | - | - |
|  | mean FD | 1.628 | 0.329 |
| ADHD-Placebo vs TD controls | group | 4.105 | <0.001*** |
|  | FSIQ | 1.874 | 0.195 |
|  | mean FD | 0.799 | 1 |
| ADHD-MPH vs TD controls | group | 1.747 | 0.255 |
|  | FSIQ | 1.602 | 0.341 |
|  | mean FD | 2.162 | 0.102 |

ADHD, attention-deficit/hyperactivity disorder; ADHD-MPH, ADHD patients under osmotic release oral system methylphenidate administration condition; ADHD-Placebo, ADHD patients under placebo condition; FD, framewise displacement; FSIQ, full scale intelligence quotient; NII, network interaction index; TD, typically-developing; ****p* < 0.001.

**Supplemental Table S3.** **Variability of time-varying NII across brain states after controlling for FSIQ and movement.**

|  |  | *t* | *p* |
| --- | --- | --- | --- |
| ADHD-MPH vs ADHD-Placebo | group | -3.108 | 0.012* |
|  | FSIQ | - | - |
|  | mean FD | 0.955 | 1 |
| ADHD-Placebo vs TD controls | group | 4.138 | <0.001*** |
|  | FSIQ | 0.926 | 1 |
|  | mean FD | 2.007 | 0.145 |
| ADHD-MPH vs TD controls | group | 1.473 | 0.435 |
|  | FSIQ | 0.052 | 1 |
|  | mean FD | 1.633 | 0.321 |

ADHD, attention-deficit/hyperactivity disorder; ADHD-MPH, ADHD patients under osmotic release oral system methylphenidate administration condition; ADHD-Placebo, ADHD patients under placebo condition; FD, framewise displacement; FSIQ, full scale intelligence quotient; NII, network interaction index; TD, typically-developing; ****p* < 0.001; **p* < 0.05.

**Supplemental Table S4. Multiple linear regression analysis revealed that variability of dynamic time-varying network interaction index (NII) was the most robust predictor of the effect of methylphenidate on sustained attention**

|  | Average change of CPT scores | | |
| --- | --- | --- | --- |
|  | (ADHD-MPH minus ADHD-Placebo) | | |
|  | *β* | *t* | *p* |
| Dynamic time-varying NII (mean) under placebo | -0.058 | 0.257 | 0.799 |
| Age | 0.373 | 1.811 | 0.084 |
| FSIQ | -0.078 | 0.345 | 0.734 |
| Mean FD under placebo | 0.002 | 0.008 | 0.994 |
| Dynamic time-varying NII (SD) under placebo | -26.570 | 2.151 | 0.043* |
| Age | 1.230 | 1.509 | 0.145 |
| FSIQ | -0.030 | 0.161 | 0.874 |
| Mean FD under placebo | 10.800 | 0.300 | 0.767 |

ADHD-MPH, children with ADHD under osmotic release oral system methylphenidate administration; ADHD-Placebo, children with ADHD under the placebo condition; CPT, continuous performance task; FD, framewise displacement; FSIQ, full scale intelligence quotient; SD, standard deviation; **p* < 0.05

**Supplemental Table S5. Euclidean distance of ADHD-MPH and ADHD-Placebo groups with TD controls for each brain state.**

|  | TD S1 | TD S2 | TD S3 |
| --- | --- | --- | --- |
| ADHD-Placebo S1 | 1.54 | 0.33 | 1.28 |
| ADHD-Placebo S2 | 0.75 | 0.98 | 0.54 |
| ADHD-MPH S1 | 1.52 | 1.13 | 0.53 |
| ADHD-MPH S2 | 1.33 | 0.21 | 1.31 |
| ADHD-MPH S3 | 0.36 | 1.06 | 0.87 |

ADHD-MPH, children with ADHD under osmotic release oral system methylphenidate administration; ADHD-Placebo, children with ADHD under the placebo condition; TD, typically developing children; S1, latent brain state 1; S2, latent brain state 2; S3; latent brain state 3.

**5. Supplemental References**

1. American Psychiatric Association. *Diagnostic and Statistical Manual of Mental Disorders: DSM-5*. (American Psychiatric Association, 2013). doi:10.1176/appi.books.9780890425596

2. Kaufman, J. *et al.* Schedule for Affective Disorders and Schizophrenia for School-Age Children-Present and Lifetime Version (K-SADS-PL): initial reliability and validity data. *J. Am. Acad. Child Adolesc. Psychiatry* **36**, 980–8 (1997).

3. Miyawaki, D., Suzuki, F., Mamoto, A., Takahashi, K., Kiriike, N. The reliability and validity of Japanese version of the schedule for affective disorders and schizophrenia for school-age children — present and lifetime version (K-SADS-PL). *Japanese J. Child Adolesc. Psychiatry* 197 (2003).

4. Sheehan, D. V *et al.* Reliability and validity of the Mini International Neuropsychiatric Interview for Children and Adolescents (MINI-KID). *J. Clin. Psychiatry* **71**, 313–326 (2010).

5. Wechsler, D. *Wechsler intelligence Scale for Children-WISC-IV*. (Psychological Corporation, 2003).

6. Conners, C. K., Pitkanen, J. & Rzepa, S. R. Conners 3rd Edition (Conners 3; Conners 2008). in *Encyclopedia of Clinical Neuropsychology* (ed. J. S. Kreutzer, J. DeLuca, B. C.) 675–678 (Springer New York, 2011). doi:10.1007/978-0-387-79948-3_1534

7. Oldfield, R. C. The assessment and analysis of handedness: The Edinburgh inventory. *Neuropsychologia* **9**, 97–113 (1971).

8. Willcutt, E. G. The prevalence of DSM-IV attention-deficit/hyperactivity disorder: a meta-analytic review. *Neurotherapeutics* **9**, 490–499 (2012).

9. Xu, G., Strathearn, L., Liu, B., Yang, B. & Bao, W. Twenty-Year Trends in Diagnosed Attention-Deficit/Hyperactivity Disorder Among US Children and Adolescents, 1997-2016. *JAMA Netw. open* **1**, e181471 (2018).

10. Hallquist, M. N., Hwang, K. & Luna, B. The nuisance of nuisance regression: spectral misspecification in a common approach to resting-state fMRI preprocessing reintroduces noise and obscures functional connectivity. *Neuroimage* **82**, 208–25 (2013).

11. Zhou, Z.-W. *et al.* Inconsistency in Abnormal Functional Connectivity Across Datasets of ADHD-200 in Children With Attention Deficit Hyperactivity Disorder. *Front. psychiatry* **10**, 692 (2019).

12. Mizuno, Y. *et al.* Catechol-O-methyltransferase polymorphism is associated with the cortico-cerebellar functional connectivity of executive function in children with attention-deficit/hyperactivity disorder. *Sci. Rep.* **7**, 4850 (2017).

13. Wilens, T. *et al.* ADHD treatment with once-daily OROS methylphenidate: final results from a long-term open-label study. *J. Am. Acad. Child Adolesc. Psychiatry* **44**, 1015–1023 (2005).

14. Akhondzadeh, S., Mohammadi, M. R. & Khademi, M. Zinc sulfate as an adjunct to methylphenidate for the treatment of attention deficit hyperactivity disorder in children: A double blind and randomized trial [ISRCTN64132371]. *BMC Psychiatry* **4**, 1–6 (2004).

15. *Concerta® Tablets (Methylphenidate Hydrochloride), Common Technical Document in Japan(October 26 2007、CTD2.7.6.8)*. (2007).

16. Huang-Pollock, C. L., Karalunas, S. L., Tam, H. & Moore, A. N. Evaluating vigilance deficits in ADHD: a meta-analysis of CPT performance. *J. Abnorm. Psychol.* **121**, 360–71 (2012).

17. Fair, D. A. *et al.* Atypical default network connectivity in youth with attention-deficit/hyperactivity disorder. *Biol. Psychiatry* **68**, 1084–1091 (2010).

18. Chen, T., Cai, W., Ryali, S., Supekar, K. & Menon, V. Distinct Global Brain Dynamics and Spatiotemporal Organization of the Salience Network. *PLoS Biol.* **14**, 1–21 (2016).

19. Zalesky, A., Fornito, A., Cocchi, L., Gollo, L. L. & Breakspear, M. Time-resolved resting-state brain networks. *Proc. Natl. Acad. Sci. U. S. A.* **111**, 10341–6 (2014).

20. Allen, E. A. *et al.* Tracking whole-brain connectivity dynamics in the resting state. *Cereb. Cortex* **24**, 663–76 (2014).

21. Charrad, M., Ghazzali, N., Boiteau, V. & Niknafs, A. Nbclust: An R package for determining the relevant number of clusters in a data set. *J. Stat. Softw.* **61**, 1–36 (2014).

22. Rashid, B. *et al.* Classification of schizophrenia and bipolar patients using static and dynamic resting-state fMRI brain connectivity. *Neuroimage* **134**, 645–657 (2016).

23. Menon, V. & Uddin, L. Q. Saliency, switching, attention and control: a network model of insula function. *Brain Struct. Funct.* **214**, 655–667 (2010).

24. Menon, V. *Salience Network*. *Brain Mapping: An Encyclopedic Reference* **2**, (Elsevier Inc., 2015).

25. Cai, W., Chen, T., Szegletes, L., Supekar, K. & Menon, V. Aberrant Time-Varying Cross-Network Interactions in Children With Attention-Deficit/Hyperactivity Disorder and the Relation to Attention Deficits. *Biol. psychiatry. Cogn. Neurosci. neuroimaging* **3**, 263–273 (2018).
